# Post-reversible cerebral vasoconstriction syndrome headache

**DOI:** 10.1186/s10194-021-01223-9

**Published:** 2021-03-25

**Authors:** Yu-Hsiang Ling, Yen-Feng Wang, Jiing-Feng Lirng, Jong-Ling Fuh, Shuu-Jiun Wang, Shih-Pin Chen

**Affiliations:** 1 Department of Neurology, Neurological Institute, Taipei Veterans General Hospital, Taipei, Taiwan; 2 School of Medicine, National Yang Ming Chiao Tung University, Taipei, Taiwan; 3 Department of Radiology, Taipei Veterans General Hospital, Taipei, Taiwan; 4 Brain Research Center, National Yang Ming Chiao Tung University, Taipei, Taiwan; 5 Division of Translational Research, Department of Medical Research, Taipei Veterans General Hospital, Taipei, Taiwan; 6 Institute of Clinical Medicine, National Yang Ming Chiao Tung University, Taipei, Taiwan

**Keywords:** Vasospasm, Chronic headache, Thunderclap headache, Prognosis, Prospective

## Abstract

**Background:**

Chronic headache may persist after the remission of reversible cerebral vasoconstriction syndrome (RCVS) in some patients. We aimed to investigate the prevalence, characteristics, risk factors, and the impact of post-RCVS headache.

**Methods:**

We prospectively recruited patients with RCVS and collected their baseline demographics, including psychological distress measured by Hospital Anxiety and Depression scale. We evaluated whether the patients developed post-RCVS headache 3 months after RCVS onset. The manifestations of post-RCVS headache and headache-related disability measured by Migraine Disability Assessment (MIDAS) scores were recorded.

**Results:**

From 2017 to 2019, 134 patients with RCVS were recruited, of whom, 123 finished follow-up interviews (response rate 91.8%). Sixty (48.8%) patients had post-RCVS headache. Migrainous features were common in post-RCVS headache. Post-RCVS headache caused moderate-to-severe headache-related disability (MIDAS score > 10) in seven (11.7%) patients. Higher anxiety level (odds ratio 1.21, *p* = 0.009) and a history of migraine (odds ratio 2.59, *p* = 0.049) are associated with post-RCVS headache. Survival analysis estimated that 50% post-RCVS headache would recover in 389 days (95% confidence interval: 198.5–579) after disease onset.

**Conclusions:**

Post-RCVS headache is common, affecting half of patients and being disabling in one-tenth. Higher anxiety level and migraine history are risk factors. Half of the patients with post-RCVS headache would recover in about a year.

## Introduction

Reversible cerebral vasoconstriction syndrome (RCVS) is a disorder with distinguishing features of abrupt, excruciating headaches (mostly thunderclap headaches, TCHs) and reversible segmental intracranial vasoconstriction revealed by neuroimaging [1]. Although most patients have a favourable outcome, RCVS remains a medical emergency due to its potential devastating complications such as ischemic stroke, intracranial haemorrhage, posterior reversible encephalopathy syndrome (PRES), and seizures.

Characterized by recurrent TCHs that are repeatedly elicited by triggers such as exertion, bathing, sexual intercourse, and Valsalva manoeuvres in a period of two to 3 weeks after disease onset, RCVS was traditionally considered as a monophasic disease that was limited to a period of 3 months. As new evidence [2, 3] has surfaced, RCVS is now recognized as a recurrent disorder with a recurrence rate of at least 5%. Also, a few studies documented RCVS victims who continued to have chronic headache after remission of RCVS bout [4, 5]. In a retrospective study [6] of a cohort from two medical centres in the United States, 53% of patients (*n* = 45) reported chronic headaches after RCVS onset. Due to the emerging awareness regarding the importance of headaches that develop after RCVS remission, criteria for “persistent headache attributed to past RCVS” were proposed in the International Classification of Headache Disorders (2018), third edition (ICHD-3, code 6.7.3.3) [7]. However, no prospective long-term study has examined the prevalence of headaches that emerge and persist after the remission of TCHs during the RCVS bout. Therefore, this study aimed to investigate the prevalence and impact of post-RCVS headache and also to identify the risk factors.

## Methods

### Participants and clinical settings

We prospectively recruited patients with RCVS from the headache clinic of Taipei Veterans General Hospital, a 2802-bed national medical centre. The study period was from January 2017 to October 2019. The diagnosis of RCVS was made according to the International Classification of Headache Disorders, third edition beta version (ICHD-3β) for those recruited before January 2018 and the International Classification of Headache Disorders, third edition (ICHD-3) for those recruited thereafter. The duration criterion that confines the clinical course of RCVS into 3 months (6.7.3.1, criterion D, ICHD-3) was omitted since the purpose of this study was to investigate the chronic headaches after RCVS.

### Diagnostic evaluations and treatments

The diagnostic procedures and interventions administered to the patients have been detailed elsewhere [3, 8, 9]. Baseline psychological distress was assessed by self-administered Hospital Anxiety and Depression Scale (HADS). Within 2 days after the patient first visited the clinic, all clinical, neuroimaging, and laboratory investigations were performed to confirm cerebral vasoconstrictions and to exclude all other possible causes of TCHs, especially aneurysmal subarachnoid haemorrhage. Patients underwent oral (30 ~ 60 mg/4 h) or intravenous (0.5 ~ 2 mg/hour) nimodipine therapy with close blood pressure monitoring immediately after the diagnosis of RCVS was made. Sequential magnetic resonance angiography (MRA) and transcranial colour-coded sonography were performed to ensure the reversibility of vasoconstrictions, after which, nimodipine was discontinued.

### Evaluation of post-RCVS headaches

In this study, a semi-structured follow-up interview focusing on post-RCVS headaches was arranged at least 3 months after RCVS onset. However, variations were allowed depending on participants’ clinical condition and availability. Patients who failed to complete the first follow-up within 6 months after RCVS onset were considered as non-respondents. Any reported headaches were defined as post-RCVS headaches. Board-certified neurologists approached our participants with a questionnaire-based, semi-structured interview, and the characteristics of post-RCVS headaches (location, intensity, accompanying symptoms, and triggers) were recorded. The interview also evaluated the impact and disability due to post-RCVS headaches, applying the Migraine Disability Assessment (MIDAS), Taiwan version [10]. Of note, to avoid the MIDAS score from being confounded by headache attacks during the RCVS acute bouts, participants were instructed to trace back until the day they had their last thunderclap headache. The grading of headache-related disability was derived from the MIDAS scores: little or no disability (MIDAS scores 0–5), mild disability (MIDAS scores 6–10), moderate disability (MIDAS scores 11–20), and severe disability (MIDAS scores > 20). Patients with post-RCVS headaches were followed in the outpatient clinic of the headache centre, Taipei Veterans General Hospital.

### Standard protocol approvals, registrations, and patient consents

This study was approved by the Institutional Review Board of Taipei Veterans General Hospital (2015–11-005C and 2019–02-013A). All participants provided written informed consent before entering the study. All clinical investigations were conducted according to the principles expressed in the Declaration of Helsinki. The corresponding author has full access to all of the data in this study and has the final responsibility for the decision to submit research for publication.

### Statistics

All analyses were performed with the SPSS software package, version 24.0 (IBM, Armonk, NY, USA). Descriptive data are presented as the mean ± standard deviation, median (range), or number (percentage). The Student’s *t* test, Fisher’s exact test, chi-squared test, or Mann-Whitney *U* test was used for comparisons between groups, when appropriate. Logistic regression models were used to determine the clinical features associated with post-RCVS headaches, and the odds ratios (ORs) of the risk factors were reported. The probability of patients being free from post-RCVS headaches over time was analyzed by Kaplan-Meyer survival curves. The 95% confidence intervals (CI) were reported. All calculated *p* values were two-tailed. Statistical significance was defined as *p* < 0.05.

### Data availability

The data that support the findings of this study are available from the corresponding author on reasonable request.

## Results

### Demographics and characteristics of the participants

**Fig. 1:**
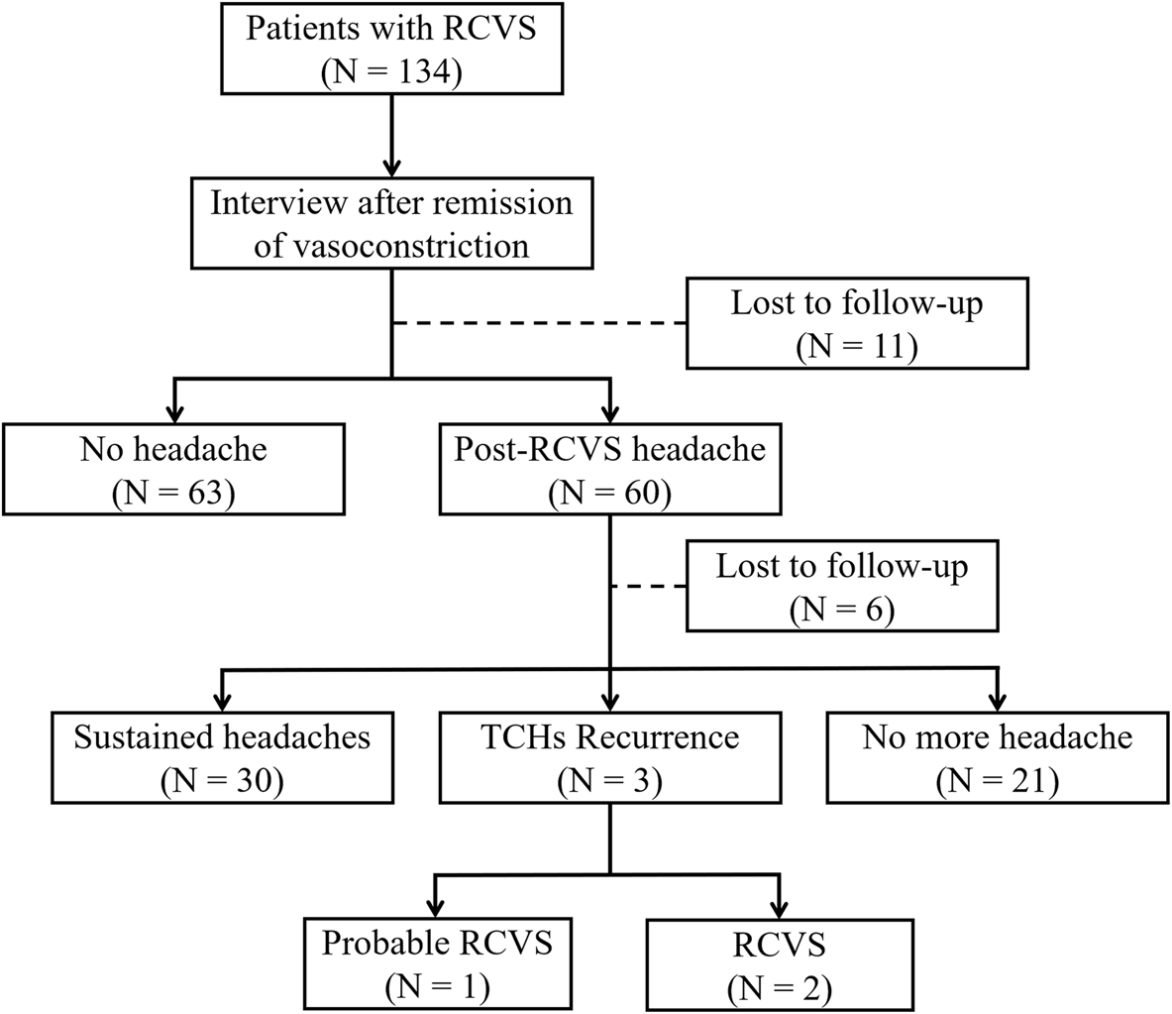
Study design and flow chart

During the study period, 134 patients fulfilled the inclusion criteria and joined the study. Of them, 123 patients completed the follow-up interviews (responder rate = 91.8%). Figure 1 shows the flow chart of this study. The demographics and characteristics of the participants are shown in Table 1. Of the patients with RCVS, five had a history of RCVS, i.e., these five patients were having recurrent RCVS bouts when they were recruited. Another four patients developed recurrent RCVS during the follow-up period.

**Table 1:** Demographics and characteristics of RCVS patients

	All RCVS (*N* = 123)	Post-RCVS headache		*P* value
		Yes (*N* = 60)	No (*N* = 63)	
Age (years)	46.6 ± 10.8	46.3 ± 9.7	46.9 ± 11.8	0.745
Sex (female)	86 (69.9)	45 (75.0)	41 (65.1)	0.230
Level of anxiety^a^	5.6 ± 3.2	6.5 ± 3.1	4.7 ± 3.0	0.004^*^
Level of depression^a^	4.3 ± 3.4	4.8 ± 3.4	3.8 ± 3.5	0.131
Past history				
Any headache	56 (45.5)	32 (53.3)	24 (38.1)	0.091
Migraine	39 (31.7)	26 (43.3)	13 (20.6)	0.007^*^
Hypertension	6 (4.9)	3 (5.0)	3 (4.8)	0.951
Diabetes Mellitus	5 (4.1)	1 (1.7)	4 (6.3)	0.189
Dyslipidemia	4 (3.3)	2 (3.3)	2 (3.2)	0.960
Triggers for TCH				
Sexual activities	54 (43.9)	23 (38.3)	31 (49.2)	0.225
Cough & Valsalva maneuvers	54 (43.9)	24 (40.0)	30 (47.6)	0.395
Exertion	29 (23.6)	15 (25.0)	14 (22.2)	0.717
Bathing	32 (26.0)	14 (23.3)	18 (28.6)	0.508
Emotion	17 (13.8)	8 (13.3)	9 (14.3)	0.878
Complications^b^	4 (3.3)	0 (0)	4 (6.3)	N/A
Convexity SAH	2	0	2	N/A
ICH	1	0	1	N/A
Ischemic stroke	1	0	1	N/A
PRES	2	0	2	N/A
Seizure	1	0	1	N/A
Possible etiologies				
Post-partum	5	3	2	N/A
Vasoactive drugs	2	1	1	N/A
History of prior RCVS	5 (4.1)	3 (5)	2 (3.2)	0.608

Data were presented as n (%) or mean ± SD. ^*^ *p* < 0.05

^a^ Levels of anxiety and depression were measured by Hospital Anxiety and Depression Scale

^b^ A patient was complicated with more than one complication

*ICH* intracerebral hemorrhage; *N/A* not applicable for calculation; *PRES* posterior reversible encephalopathy syndrome; *RCVS* reversible cerebral vasoconstriction syndrome; *SAH* subarachnoid hemorrhage; *TCH* thunderclap headache

### Patients with post-RCVS headache

Overall, 60 (48.8%) participants reported post-RCVS headaches. There were no differences in age, sex, or triggers that elicited TCHs between patients with and without post-RCVS headaches. Patients with post-RCVS headaches had a higher level of anxiety, i.e., higher mean anxiety score of HADS at the first presentation to the clinic (Table 1). Compared to those without post-RCVS headache, the presence of a history of migraine prior to RCVS onset was more common in patients with post-RCVS headaches. The frequency of hypertension, diabetes mellitus or dyslipidaemia in patients with post-RCVS headaches did not differ from those without. Of note, two (3.3%) patients had suffered from daily persistent headaches since the onset of TCHs during RCVS acute bouts.

### Characteristics of post-RCVS headache

The characteristics of post-RCVS headache are summarized in Table 2. The headaches were frequently located in the occipital area (45.0%), temporal area (41.7%), and vertex (30.0%). Migrainous features were not uncommon in post-RCVS headaches. Of the 60 patients with post-RCVS headaches, 31.7% reported unilaterality, 44.1% reported pulsatile headache, and 25.0% reported that their headaches were aggravated by physical activities. Regarding the accompanying symptoms, 20.3% reported nausea, 10.0% reported vomiting, 16.7% reported photophobia, and 43.3% reported phonophobia. Overall, 12 (20%) patients had post-RCVS headaches fulfilling the features of ICHD-3 migraine.

**Table 2:** Headache features of post-RCVS headaches

	Post RCVS headache (*N* = 60)
Location	
Frontal	7 (11.7)
Temporal	25 (41.7)
Vertex	18 (30.0)
Occipital	27 (45.0)
Whole head	5 (8.3)
Headache intensity^a^	3.7 ± 2.6
Migrainous features	
Unilateral	19 (31.7)
Pulsating	26 (44.1)
Aggravated by physical activity	15 (25.0)
Nausea	12 (20.3)
Vomiting	6 (10.0)
Photophobia	10 (16.7)
Phonophobia	26 (43.3)
Fulfill ICHD-3 criteria	
Migraine	12 (20.0)
Disability^b^	
Little or no disability	47 (78.3)
Mild disability	6 (10.0)
Moderate disability	1 (1.7)
Severe disability	6 (10.0)
Triggers	
None	35 (58.3)
Sexual activities	5 (8.3)
Cough & Valsalva maneuvers	9 (15.0)
Exertion	5 (8.3)
Bathing	4 (6.7)
Emotion	20 (33.3)

Data were presented as n (%), mean ± SD

^a^ Headache intensity was measured with an 11-point numeric rating scale where 0 represents no pain at all and 10 represents the worst imaginable pain

^b^ Disability was measured by the Migraine Disability Assessment

*ICHD-3* The International Classification of Headache Disorders, 3rd Edition; *RCVS* reversible cerebral vasoconstriction syndrome

The post-RCVS headaches were mostly mild (*n* = 37, 61.7%), while the rest were moderate (*n* = 18, 30.0%) or severe (*n* = 5, 8.3%). The average intensity score on a scale from zero to ten was 3.7 ± 2.6. Regarding the MIDAS score, most patients reported little or no disability; however, 11.7% reported that their post-RCVS headaches caused moderate-to-severe disability. Despite the fact that none of the analysed post-RCVS headaches were TCHs, 25 (41.7%) patients reported at least one headache trigger, including sexual activity (8.3%), cough and other Valsalva manoeuvres (15.0%), exertion (8.3%), bathing (6.7%), and emotion (33.3%) (Table 2).

### Factors associated with post-RCVS headaches

Clinical features, including demographics, prior medical history, and triggers for TCHs as well as accompanying symptoms in RCVS were analyzed to identify predictors for post-RCVS headaches using logistic regression models (Table 3). The univariate analyses showed that having a prior history of migraine and a higher level of anxiety were associated with the presence of post-RCVS headaches. These factors remained significant according to a multivariable analysis (history of migraine: OR = 2.59, 95% CI 1.01 ~ 6.69, *p* = 0.049; anxiety level: OR = 1.21, 95% CI 1.05 ~ 1.39, *p* = 0.009).

**Table 3:** Factors associated with post-RCVS headache

	Univariate		Multivariable	
	OR (95% CI)	*P* value	OR (95% CI)	*P* value
Past medical history				
Any headache	1.76 (0.85, 3.65)	0.130		
Migraine	2.79 (1.25, 6.23)	0.013^*^	2.59 (1.001, 6.69)	0.049^*^
Hypertension	1.08 (0.21, 5.59)	0.931		
Diabetes mellitus	0.27 (0.29, 2.54)	0.253		
Dyslipidemia	1.12 (0.15, 8.45)	0.913		
Level of anxiety^a^	1.21 (1.05, 1.40)	0.007^*^	1.21 (1.05, 1.39)	0.009^*^
Level of depression^a^	1.08 (0.96, 1.22)	0.188		
Triggers for TCHs				
Sexual activities	0.65 (0.24, 1.79)	0.406		
Cough & Valsalva maneuvers	0.64 (0.30, 1.36)	0.242		
Exertion	1.27 (0.54, 2.97)	0.584		
Bath	0.71 (0.30, 1.70)	0.445		
Emotion	0.80 (0.28, 2.29)	0.674		

The results were controlled by age and sex. ^*^ *p* < 0.05; ^a^ Levels of anxiety and depression were measured by Hospital Anxiety and Depression Scale

*RCVS* reversible cerebral vasoconstriction syndrome; *OR* odds ratio; *CI* confidence interval; *TCHs* thunderclap headaches

### Follow-ups of post-RCVS headaches

**Fig. 2:**
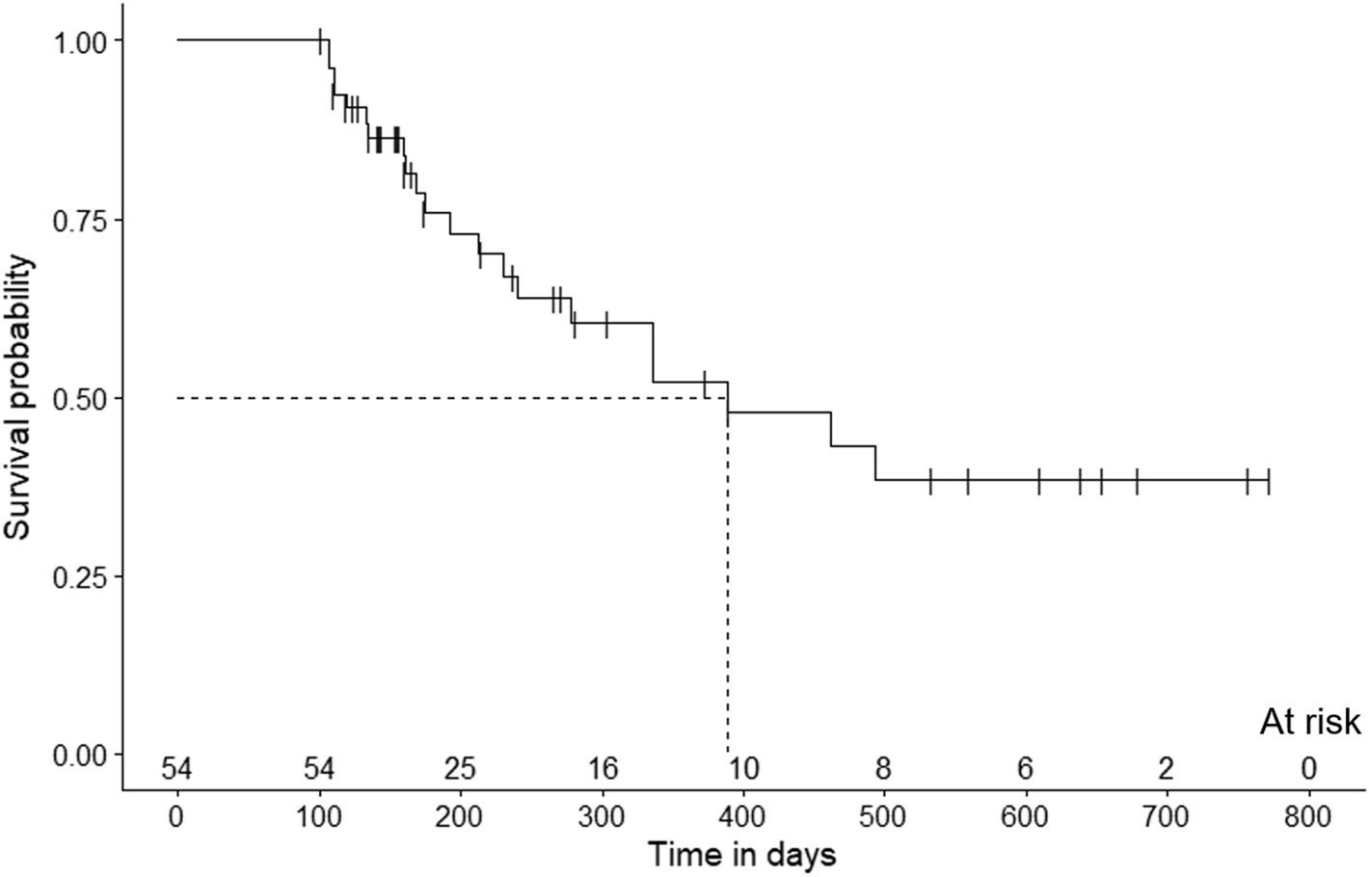
The survival curve of remission of post-RCVS headache

Fifty-four (90%) patients with post-RCVS headaches were followed at the outpatient clinic after the interview. The mean follow-up duration was 271.1 ± 186.8 days after their first visits. Thirty (55.6%) patients continued to have headaches, with the most extended follow-up duration up to 758 days (Fig. 2). The median headache-free probability of post-RCVS headache was 389.0 days (95% CI: 198.5–579 days). Post-RCVS headaches resolved in 21 patients (38.9%). Three patients developed thunderclap headaches during the clinical follow-ups. Two of them were confirmed to have RCVS relapse based on MRAs. The one without apparent vasoconstriction was diagnosed with probable RCVS after excluding other etiologies.

## Discussion

In this prospective longitudinal follow-up study, about half of our RCVS participants still suffered from chronic headaches at 3 months after RCVS onset. Half of them (30/60, 50%) required long-term medical attention, lasting up to 2 years. Furthermore, we discovered that the RCVS patients with a history of migraine and a higher anxiety level were at risk for post-RCVS headaches. To the best of our knowledge, this is the first prospective and systematic study investigating the frequency and risk factors of headaches after RCVS.

We found that headaches after RCVS onset were common and mostly mild in intensity as well as headache-related disability, consistent with the finding of a previous retrospective [6] study that recruited 45 patients from various ethnicities. However, post-RCVS headaches caused moderate to severe disability in 10% of patients, who required chronic medical attention. In addition, we identified two patients (3.3%) who suffered from daily persistent headaches since RCVS onset, which was in line with previous reports demonstrating five cases presenting with new daily persistent headaches after RCVS onset [4, 5].

Our study found that having a history of migraine is a risk factor that predicts post-RCVS headaches. Having a prior history of migraine is not unusual for patients with RCVS; in our prior cohort with 210 RCVS patients, 21% of patients had a history of migraine [3]. Also, migrainous features are not uncommon in post-RCVS headache. Up to 78.3% (47/60) of patients had at least one migrainous feature of their headaches after RCVS. These findings suggested a connection linking migraine, RCVS, and post-RCVS headache, which has an impact on both clinical and pathophysiological aspects. First, when treating these patients, especially when they develop post-RCVS headaches, physicians should be cautious about the administration of triptans and ergots since their potential vasoactive side effects on intracranial arteries. Second, whether migraine is a potential risk factor for RCVS is still under debate; however, shared mechanisms, including endothelial cell dysfunction, linking migraine and RCVS have been proposed by previous studies [11–14]. In addition, it has been shown that patients with migraine have an altered functional connectivity in pain-processing areas, including the periaqueductal grey area [15], rostral anterior cingulate and prefrontal cortex [16, 17], and insula [18]. We hypothesize that during the acute bouts of RCVS, the trigeminovascular nociceptive pathway is activated and sensitized due to the repeated attack of thunderclap headaches, causing chronic headaches after the acute phase of RCVS, especially in patients with migraine whose pain perception networks might have already been altered. We also identified that the anxiety level on the first presentation to clinics was associated with the occurrence of post-RCVS headaches. From the past studies, we learned that pain perception may be exacerbated by higher anxiety status, probably through altering the activity of the hippocampal network [19, 20], which may explain our findings.

The pathophysiology of RCVS remains unknown; thus, demystifying the mechanism of post-RCVS headache will be even more challenging. However, studies focusing on RCVS pathophysiology have given us some hints. First, a study conducted by a Korean research team demonstrated that patients with RCVS have impaired cerebral endothelial functions, and, in some patients, the endothelial cell dysfunction had not recovered at the three-month follow-up [14]. Moreover, our previous study [21] assessing the autonomic function by analyzing heart rate variability in RCVS patients demonstrated that RCVS patients had autonomic dysfunction during acute bouts, and it remained abnormal compared with healthy controls even after the remission of RCVS. These findings suggest that some patients remain abnormal after RCVS remission, providing a plausible physiological basis for post-RCVS headache. It is also possible that in vulnerable patients, their pain matrix is sensitized by TCHs or the biological consequences of RCVS, such as disruption to the blood-brain barrier. A prospective, cohort study is warranted to elucidate the causality and differentiate whether these findings resulted from the innate biological traits of RCVS patients or subclinical sequelae after RCVS remission as we suspected.

Our study has limitations. First, we assessed the baseline anxiety levels of patients on their first presentation to clinics. It is difficult to distinguish whether the anxiety we referred to represents trait anxiety or state anxiety at evaluation. We believe that state anxiety may play an essential role because the HADS required the participants to answer the questions based on their condition in the past week, and all patients had just recently suffered from TCHs, which could be considered as major and devastating events. However, it is difficult to entirely exclude the possibility that the trait anxiety of a patient confounded the anxiety score we measured. In fact, it is likely that the anxiety level we measured in this study represented both the state and trait anxiety. Second, since all patients were treated initially with nimodipine, the effect of nimodipine on the occurrence of post-RCVS headaches could not be evaluated in this study. Third, it is methodologically challenging to distinguish post-RCVS headache from migraine attacks in patients with pre-existing migraine. In certain cases, it is almost impossible for physicians, or even the patients themselves, to determine the differences between a pre-existing migraine and a chronic headache after RCVS. Following the context, diagnosing a migraine-like headache as post-RCVS headache is not against the principles of ICHD criteria. On the other hand, post-RCVS headache may not be completely equivalent to migraine even when it fulfils the diagnostic criteria of migraine since some common features in post-RCVS headache, like having headache triggers including sex activities, Valsalva-like manoeuvres, bathing, and emotional changes, were not seen in migraine. Finally, we believe that post-RCVS headaches should be distinguished from migraine since we are wary of using abortive or preventive medications that are potentially vasoconstrictive in patients with post-RCVS headache.

## Data Availability

The data that support the findings of this study are available from the corresponding author on reasonable request.

## Abbreviations

CI: Confident interval
HADS: Hospital Anxiety and Depression Scale
ICHD: International classification of headache disorder
MIDAS: Migraine Disability Assessment
MRA: Magnetic resonance angiography
OR: Odds ratio
RCVS: Reversible cerebral vasoconstriction syndrome
TCH: Thunderclap headache

## References

[1] Calabrese LH, Dodick DW, Schwedt TJ, Singhal AB (2007). Narrative review: reversible cerebral vasoconstriction syndromes. Ann Intern Med.

[2] Boitet R, de Gaalon S, Duflos C, Marin G, Mawet J, Burcin C, Roos C, Fiedler U, Bousser MG, Ducros A (2020). Long-term outcomes after reversible cerebral vasoconstriction syndrome. Stroke.

[3] Chen S-P, Fuh J-L, Lirng J-F, Wang YF, Wang SJ (2015). Recurrence of reversible cerebral vasoconstriction syndrome: a long-term follow-up study. Neurology.

[4] Rozen TD, Beams JL (2013). New daily persistent headache with a thunderclap headache onset and complete response to nimodipine (a new distinct subtype of NDPH). J Headache Pain.

[5] Jamali SA, Rozen TD (2019). An RCVS Spectrum disorder? New daily persistent headache starting as a single thunderclap headache (3 new cases). Headache.

[6] John S, Singhal AB, Calabrese L, Uchino K, Hammad T, Tepper S, Stillman M, Mills B, Thankachan T, Hajj-Ali RA (2016). Long-term outcomes after reversible cerebral vasoconstriction syndrome. Cephalalgia.

[7] (2018) Headache Classification Committee of the International Headache Society (IHS) The International Classification of Headache Disorders, 3rd edition. Cephalalgia 38:1–211. 10.1177/0333102417738202 10.1177/0333102417738202 29368949

[8] Chen S-P, Fuh J-L, Wang S-J, Chang FC, Lirng JF, Fang YC, Shia BC, Wu JC (2010). Magnetic resonance angiography in reversible cerebral vasoconstriction syndromes. Ann Neurol.

[9] Chen S-P, Chou K-H, Fuh J-L, Huang YH, Huang CC, Lirng JF, Wang YF, Lin CP, Wang SJ (2018). Dynamic changes in white matter Hyperintensities in reversible cerebral vasoconstriction syndrome. JAMA Neurol.

[10] Hung P-H, Fuh J-L, Wang S-J (2006) Validity, reliability and application of the Taiwan version of the migraine disability assessment questionnaire. J Formos Med Assoc 105:563–568. 10.1016/S0929-6646(09)60151-0, 7 10.1016/S0929-6646(09)60151-0 16877236

[11] Lee S-T, Chu K, Jung K-H (2008). Decreased number and function of endothelial progenitor cells in patients with migraine. Neurology.

[12] Rodríguez-Osorio X, Sobrino T, Brea D (2012). Endothelial progenitor cells: a new key for endothelial dysfunction in migraine. Neurology.

[13] Chen S-P, Wang Y-F, Huang P-H, Chi CW, Fuh JL, Wang SJ (2014). Reduced circulating endothelial progenitor cells in reversible cerebral vasoconstriction syndrome. J Headache Pain.

[14] Choi HA, Lee MJ, Chung C-S (2017). Cerebral endothelial dysfunction in reversible cerebral vasoconstriction syndrome: a case-control study. J Headache Pain.

[15] Mainero C, Boshyan J, Hadjikhani N (2011). Altered functional magnetic resonance imaging resting-state connectivity in periaqueductal gray networks in migraine. Ann Neurol.

[16] Xue T, Yuan K, Cheng P, Zhao L, Zhao L, Yu D, Dong T, von Deneen KM, Gong Q, Qin W, Tian J (2013). Alterations of regional spontaneous neuronal activity and corresponding brain circuit changes during resting state in migraine without aura. NMR Biomed.

[17] Yu D, Yuan K, Zhao L, Zhao L, Dong M, Liu P, Wang G, Liu J, Sun J, Zhou G, Deneen KM, Liang F, Qin W, Tian J (2012). Regional homogeneity abnormalities in patients with interictal migraine without aura: a resting-state study. NMR Biomed.

[18] Xue T, Yuan K, Zhao L, Yu D, Zhao L, Dong T, Cheng P, von Deneen KM, Qin W, Tian J (2012). Intrinsic brain network abnormalities in migraines without aura revealed in resting-state fMRI. PLoS One.

[19] Tang J, Gibson SJ (2005). A psychophysical evaluation of the relationship between trait anxiety, pain perception, and induced state anxiety. J Pain.

[20] Ploghaus A, Narain C, Beckmann CF, Clare S, Bantick S, Wise R, Matthews PM, Rawlins JNP, Tracey I (2001). Exacerbation of pain by anxiety is associated with activity in a hippocampal network. J Neurosci.

[21] Chen S-P, Yang AC, Fuh J-L, Wang S-J (2013). Autonomic dysfunction in reversible cerebral vasoconstriction syndromes. J Headache Pain.

